# Identification of a novel autoantibody against self-vimentin specific in secondary Sjögren’s syndrome

**DOI:** 10.1186/s13075-017-1508-5

**Published:** 2018-02-12

**Authors:** Yu-Hui Li, Ya-Ping Gao, Jie Dong, Lian-Jie Shi, Xiao-Lin Sun, Ru Li, Xue-Wu Zhang, Yu Liu, Li Long, Jing He, Qun-Jie Zhong, Eric Morand, Guang Yang, Zhan-Guo Li

**Affiliations:** 1 Department of Rheumatology & Immunology, Peking University People’s Hospital, 11 Xizhimen South Street, Beijing, 100044 China; 2 Beijing Institute of Basic Medical Sciences, Beijing, China; 3 Arthritis Clinic and Research Center, Peking University People’s Hospital, Beijing, China; 4 Center for Inflammatory Diseases, Southern Clinical School, Monash University, Melbourne, Australia

**Keywords:** Sjögren’s syndrome, Biomarker, Autoantigen, Vimentin

## Abstract

**Background:**

Sjögren’s syndrome (SS) is a primary autoimmune disease (pSS) or secondarily associated with other autoimmune diseases (sSS). The mechanisms underlying immune dysregulation in this syndrome remain unknown, and clinically it is difficult to diagnose owing to a lack of specific biomarkers.

**Methods:**

We extracted immunoglobulins (Igs) from the sera of patients with sSS associated with rheumatoid arthritis (RA) and used them to screen a phage display library of peptides with random sequences.

**Results:**

Our results show that an sSS-specific peptide, designated 3S-P, was recognized by sera of 68.2% (60 of 88) patients with sSS, 66.2% of patients with RA-sSS, and 76.5% of patients with systemic lupus erythematosus (SLE)-sSS. The anti-3S-P antibody was scarcely found in patients with pSS (1.8%), RA (1.3%), SLE (4.2%), ankylosing spondylitis (0%), and gout (3.3%), as well as in healthy donors (2%). The 3S-P-binding Igs (antibodies) were used to identify antigens from salivary glands and synovial tissues from patients with sSS. A putative target autoantigen expressed in the synovium and salivary gland recognized by anti-3S-P antibody was identified as self-vimentin.

**Conclusions:**

This novel autoantibody is highly specific in the diagnosis of sSS, and the underlying molecular mechanism of the disease might be epitope spreading involved with vimentin.

**Electronic supplementary material:**

The online version of this article (doi:10.1186/s13075-017-1508-5) contains supplementary material, which is available to authorized users.

## Background

Sjögren’s syndrome (SS) is a systemic autoimmune disease characterized by marked reduction of exocrine glandular secretions, and it progresses to lymphocytic infiltration and destruction of exocrine glands [1–3]. In addition to arising as a primary disease (primary Sjögren’s syndrome [pSS]), pSS occurs in association with other autoimmune diseases (secondary Sjögren’s syndrome [sSS]), particularly rheumatoid arthritis (RA) and systemic lupus erythematosus (SLE) [4–7]. The impact of sSS on RA is illustrated by a twofold increased risk of non-Hodgkin’s lymphoma compared with patients with RA without sSS, and there is a tendency toward increased mortality in patients with RA with sSS compared with those without sSS [8–10]. Patients with SLE and SS more commonly had renal, peripheral vascular, and musculoskeletal damage than those with SLE without SS [11].

Evidence indicates that the clinical and immunological features of patients with sSS differ from those of patients with pSS [12]. Antinuclear antibody, anti-SSA, and anti-SSB have been applied to the diagnosis of SS, but none of these autoantibodies provides sufficient specificity and sensitivity [13–15]. The molecular mechanism of sSS is unknown, and the lack of specific biomarkers constrains efforts to establish the clinical diagnosis of and intervention for the disease. Therefore, the aim of the present study was to identify potential biomarkers and molecules that are specifically associated with sSS. We employed a strategy successfully applied to collagen-induced arthritis, autoimmune pancreatitis, and other autoimmune diseases using a library of peptides with random sequences [16–19]. We report the identification of a novel autoantibody that was specifically associated with sSS and recognized vimentin as an autoantigen of the disease.

## Methods

### Participants

We obtained serum samples from patients and healthy control subjects (HCs) between January 2004 and June 2011 at the People’s Hospital of Peking University. Patients diagnosed with sSS fulfilled the 2002 classification criteria for sSS [20]. Immunological data collected from medical files included the presence of antinuclear antibodies, anti-SSA and anti-SSB antibodies, rheumatoid factor, as well as other antibodies such as anti-double-stranded DNA. Blood samples were obtained after subjects provided written informed consent, and the local ethics committee approved the study. All serum samples were stored at −20 °C. For the serological antigen selection procedure, immunoglobulins (Igs) were pooled from serum samples of ten patients with sSS with RA (all patients fulfilled the 2010 classification criteria for RA) [21], ten patients with RA without SS, and ten HCs. For further validation of the identified markers, plasma samples were obtained from 88 patients with sSS (71 with RA and 17 with SLE), 99 HCs, 55 patients with pSS, 79 patients without sSS but with RA, 24 patients with SLE, 21 patients with ankylosing spondylitis, and 30 patients with gout. The characteristics of patients with sSS and HCs included in the study are summarized in Table 1 and Additional file 1: Tables S1–S3.

**Table 1:** Clinical characteristics of patients

Diagnosis	No. of subjects	Mean age, years (range)	Female/male sex (no.)
RA-sSS	71	54.2 (27–84)	64/7
SLE-sSS	17	47.3 (23–70)	15/2
RA	79	55.9 (26–77)	73/6
pSS	55	55.3 (21–79)	52/3
SLE	24	36.3 (20–75)	21/3
AS	21	41.0 (18–59)	12/9
Gout	30	54.4 (35–80)	27/3
HC	99	43.4 (22–59)	89/10

*Abbreviations: AS* Ankylosing spondylitis, *HC* Healthy control subject, *pSS* Primary Sjögren’s syndrome, *RA* Rheumatoid arthritis, *SLE* Systemic lupus erythematosus, *SS* Sjögren’s syndrome, *sSS* Secondary Sjögren’s syndrome

### Peptide library

A random dodecamer peptide library that expresses peptides on a phage virion was purchased from New England Biolabs (Ipswich, MA, USA). The peptide library was screened against three pooled IgG fractions. Each fraction was purified from the pooled sera of ten patients with RA-sSS. To enrich for specific binding phage clones (putatively disease-related), IgGs from ten patients with RA and ten HCs were employed to subtract nonspecific binding clones. Aliquots of 2 × 10^11^ plaque-forming units in 0.05% PBS with Tween 20 (PBST) were added to IgG-coated wells of a polyvinyl chloride microtiter plate and incubated for 60 minutes at room temperature. The wells were washed ten times with 0.1% PBST, and bound phages were eluted with acidic elution buffer (20 mmol/L Gly-HCl, pH 2.2). Low-pH eluate was immediately neutralized with 1 mol/L Tris-HCl, pH 9.1. Eluates were amplified by infecting *Escherichia coli* ER2738 host cells (New England Biolabs). After 4.5 h of vigorous shaking at 37 °C, bacteria were removed by centrifugation. Phage particles were purified by two consecutive precipitations with polyethylene glycol (PEG)/NaCl (20% PEG-8000, 2.5 mol/L NaCl) and then resuspended in PBS. These amplified eluates were titered to determine phage concentration and then used as the input phage for the next selection round. Finally, unamplified eluate from the third round of biopanning was used to infect plated bacterial host cells. Single phage clones were assayed by enzyme-linked immunosorbent assay (ELISA). DNA was extracted from positive clones and sequenced.

### Analysis of antibody binding

Antibody reactivity against peptides was measured using a solid-phase ELISA. Synthetic purified peptides were coated overnight at 4 °C at 2 μg/well in 0.1 M sodium hydrogen carbonate buffer, pH 9.6. Unbound antibodies were discarded, and wells were blocked with 5% bovine serum albumin (BSA) for 1 h at 37 °C. Serum samples diluted 1:25 in 1% BSA were added to the wells and incubated for 1 h. After a washing step with PBS containing 0.5% (vol/vol) Tween 20, antibody binding was detected by incubation with a goat antihuman IgG horseradish peroxidase-conjugated antibody, followed by color development using 3,3′,5,5′-tetramethylbenzidine as a substrate. The reaction was stopped by adding 1 mol/L H_2_SO_4,_ and the color intensity was measured at 450 nm. Absorbance values greater than 3 SDs above the mean for each serum dilution of the control group were considered positive.

### Preparation of polyclonal anti-3S-P antibodies

The peptide YSLHNAGPWSLHQ, designated 3S-P (secondary Sjögren’s syndrome-related peptide), was manually synthesized using a standard method [19] for solid-phase peptide synthesis. Polyclonal antibodies against the 3S-P were generated in New Zealand white rabbits using standard techniques (R-anti-3S-P) [22]. R-anti-3S-P antibodies were purified using an immunoaffinity column, which was prepared by conjugating the peptide to SulfoLink Coupling Resin (Pierce Biotechnology/Thermo Fisher Scientific, Rockford, IL, USA) according to the manufacturer’s instructions [23].

### Human synovial tissue, salivary gland biopsies, and cells

Synovial tissue specimens and fluid were obtained from patients with RA-sSS during joint replacement surgery at People’s Hospital Peking University in China. Salivary gland specimens were obtained from patients with sSS during salivary gland biopsies. All the methods were carried out in accordance with the guidelines and regulations issued by the Ethics Committee of People’s Hospital Peking University. Human primary fibroblast-like synoviocyte (FLS) cultures were established as previously described with some modifications [24]. Briefly, tissue pieces were minced, treated with 1 mg/ml collagenase for 1 h at 37 °C, and passed through a cell strainer. The cell suspension was incubated at 37 °C in complete DMEM in an atmosphere containing 5% CO_2_. Cells were harvested at passages 3 through 6.

### Immunohistochemistry

Sections of the synovial or salivary gland were mounted on glass slides. Immunohistochemical analysis was performed as previously described [25]. R-anti-3S-P and anti-keyhole limpet hemocyanin (anti-KLH) antibodies were used as primary antibodies (25 μg/ml each). The sections were subsequently stained with horseradish peroxidase-conjugated antirabbit antibody (Abcam, Cambridge, UK). Detection was accomplished using diaminobenzidine, and sections were counterstained with hematoxylin.

### Immunofluorescence and confocal microscopy

FLSs obtained from patients with RA were immobilized, fixed, and permeabilized as previously described [21]. Cells were incubated with 1% BSA for 30 minutes before incubation. The fixed cells were incubated with the purified antibodies (25 μg/ml each) for 60 minutes. After a washing step, the cells were incubated with fluorescein isothiocyanate-conjugated goat antihuman IgG diluted 1:200, and nuclei were stained with 4′,6-diamidino-2-phenylindole.

### Western blot analysis

Proteins extracted from FLSs were electrophoresed and transferred onto a Hybond ECL nitrocellulose membrane (GE Healthcare Life Sciences, Marlborough, MA, USA). The membranes were incubated with affinity-purified polyclonal R-anti-3S-P antibodies (5 μg/ml) and then with horseradish peroxidase-conjugated goat antirabbit IgG.

### Two-dimensional gel electrophoresis

Proteins obtained from FLSs were separated by two-dimensional gel electrophoresis. Protein samples were loaded onto immobilized pH gradient gel strips (GE Healthcare Life Sciences). After rehydration for 14 h, isoelectric focusing (IEF) was carried out for 1 h at 200 V, 1 h at 500 V, and 1 h at 1000 V continuously, then a gradient was applied from 1000 to 8000 for 1 h and finally at 8000 V for 8 h to reach a total of 72 KVh at 20 °C. Following IEF separation, gel strips were incubated in equilibration buffer (50 mM Tris-HCl, pH 8.8, 6 M urea, 30% glycerol, 2% sodium dodecyl sulfate [SDS]) with 10 mg/ml dithiothreitol for 15 minutes, followed by equilibration buffer with 25 mg/ml iodoacetamide for 15 minutes. Then strips were loaded onto 12.5% SDS-PAGE gels and electrophoresed for 20 minutes at a constant current of 10 mA and then at 30 mA per gel until the bromophenol blue reached the bottom of the gels. Subsequently, the two-dimensional gel was silver-stained and scanned. The digital images were analyzed using the GS-800 image scanner (Bio-Rad Laboratories, Hercules, CA, USA) at 300-dpi resolution. Spot detection, quantification, and analyses of two-dimensional protein patterns were done using PDQuest 2-D Analysis software version 7.2 (Bio-Rad Laboratories). Then the report of quantitative differences between two gel images was generated. The *t* test was performed to compare the relative volume of spots in gels. Significant spots were selected for protein identification.

### MS analysis and data interpretation

The bands of interest were excised from the gels and digested with trypsin. The trypsin-digested samples were initially transferred with an aqueous 0.1% formic acid solution to the precolumn at a flow rate of 5 μl/minute for 4 minutes. Mobile phase A was water with 0.1% formic acid, and mobile phase B was 0.1% formic acid in acetonitrile. The peptides were separated with a linear gradient of 10–40% mobile phase B over 60 minutes at 200 nl/minute, followed by 10 minutes at 85% mobile phase B. The column was re-equilibrated at initial conditions for 10 minutes. The column temperature was maintained at 35 °C. The lock mass was delivered from the auxiliary pump of the nanoACQUITY pump (Waters, Milford, MA, USA) with a constant flow rate of 400 nl/minute at a concentration of 100 fmol/μl [Glu1]-fibrinopeptide B. Analysis of trypsin peptides was performed using a SYNAPT high-definition mass spectrometer (Waters). For all measurements, the mass spectrometer was operated in V-mode with a typical resolving power of at least 10,000 FWHM. The time-of-flight (TOF) analyzer of the mass spectrometer was calibrated with the MS/MS fragmentations of [Glu1]-fibrinopeptide B from 50 to 1600 mass/charge ratio. The reference sprayer was sampled with a frequency of 30 seconds. Accurate LC-MS and MS/MS data were collected in high definition data-directed analysis mode. LC-MS/MS data were processed using ProteinLynx Global SERVER version 2.3 (PLGS 2.3; Waters), and the resulting peak lists were subjected to a search against the National Center for Biotechnology information (NCBI) protein database of with the Mascot search engine.

### Immunoprecipitation

The mouse antivimentin antibody was incubated with the FLS protein extract (400 μl) for 3 h at 4 °C. Next, protein G-conjugated magnetic beads (100 μl) were added and incubated for 1 h at 4 °C, and the beads were collected and washed as instructed by the manufacturer. Laemmli sample buffer was added next, and the mixture was heated for 5 minutes at 95 °C. The supernatants were collected after centrifugation and subjected to SDS-PAGE and Western blot analysis. To detect the 3S-P antigen, the anti-3S-P serum was diluted to 1:800, and peroxidase-conjugated goat antirabbit IgG was used as the secondary antibody. An antivimentin antibody was used at 1:1000 dilution.

### Statistical analyses

We evaluated the sensitivity and specificity of the tests using the ROC curve and estimated the AUC at 95% CIs. Statistical analysis was performed using SPSS software version 16 (SPSS, Chicago, IL, USA).

## Results

### Identification of a peptide specifically recognized by sera from patients with sSS

**Fig. 1:**
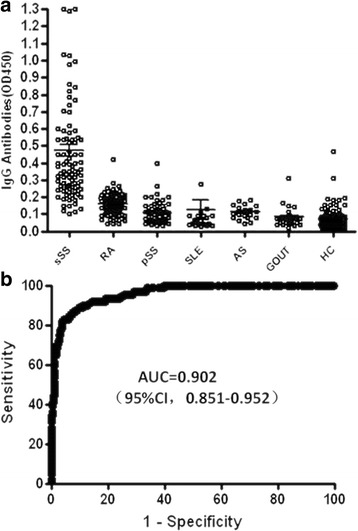
Antibodies to 3S-P in serum samples from patients and healthy control subjects. **a** ELISA results of detection of anti-3S-P IgG in the subjects’ sera. Each *circle* represents one patient. **b** Sensitivity and specificity of the assay of IgG antibodies against 3S-P for differentiating between patients with sSS and control subjects. The ROC curve indicates the level of antibodies against 3S-P in patients with sSS compared with that in patients with RA, pSS, or SLE. *sSS* Secondary Sjögren’s syndrome associated with rheumatoid arthritis, *RA* Rheumatoid arthritis, *pSS* Primary Sjögren’s syndrome, *SLE* Systemic lupus erythematosus, *AS* Ankylosing spondylitis, *HC* Healthy control subjects, *3S-P* Secondary Sjögren’s syndrome-associated peptide, *IgG* Immunoglobulin G

We screened a phage display library of random peptides using pooled protein A affinity-purified and dialyzed IgGs from ten patients with RA-sSS for specific binding to peptides. Phage clones with the sequence YSLHNAGPWSLQ specifically bound to the IgGs of patients with RA-sSS, which was confirmed by three rounds of screening with the same pooled IgGs. The dodecamer peptide was designated as 3S-P. Further investigation revealed that this peptide was recognized by sera of 68.2% (60 of 88) patients with sSS (66.2% of patients with RA-sSS and 76.5% of patients with SLE-sSS) (Fig. 1a). In contrast, anti-3S-P was barely detectable in patients with pSS, other those with rheumatic diseases, and HCs. The prevalence rates were 1.8% (1 of 55) in pSS, 1.3% (1 of 79) in RA, 4.2% (1 of 24) in SLE, 3.3% (1 of 30) in gout, and 2% (2 of 99) in HCs, respectively. We did not detect reactivity of 3S-P with sera from 21 patients with ankylosing spondylitis (0% [0 of 21]). The sensitivity and specificity of detection of anti-3S-P in sera of patients with sSS (RA-sSS and SLE-sSS), with a cutoff absorbance value of 0.29 at 450 nm, were 68.2% and 98.1%, respectively. The AUC of the ROC was 0.902 (95% CI 0.851–0.952; *P* < 0.001) (Fig. 1b).

### Autoantigen is expressed in synovial membrane and salivary gland

**Fig. 2:**
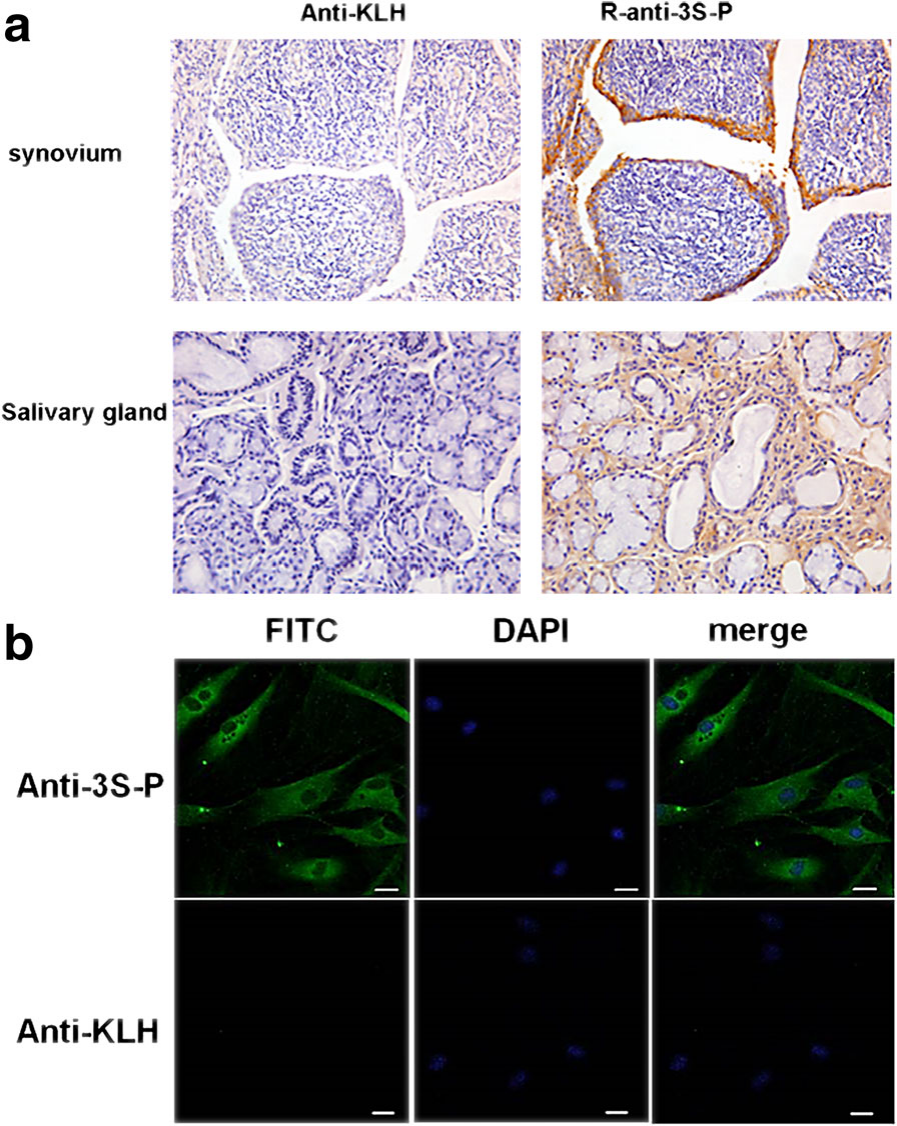
Immunohistochemical analysis of tissues and cells using anti-3S-P antibodies. **a** Expression of anti-3S-P target antigens in RA-sSS synovial tissue and salivary tissue. RA-sSS synovium was stained using antibodies against KLH and R-anti-3S-P. RA-sSS salivary gland tissue was stained using R-anti-3S-P and anti-KLH as control (original magnification × 400). **b** Confocal immunofluorescence microscopic images of FLSs reacted with R-anti-3S-P or anti-KLH antibody. Antibody staining (FITC, *green*), and nuclear staining (DAPI, *blue*) are shown. Scale bar represents 20 μm. *3S-P* Secondary Sjögren’s syndrome-associated peptide, *DAPI* 4′6-Diamidino-2-phenylindole, *FITC* Fluorescein isothiocyanate, *FLS* Fibroblast-like synoviocyte, *KLH* Keyhole limpet hemocyanin, *RA* Rheumatoid arthritis, *R-anti-3S-P* Rabbit-anti-secondary Sjögren’s syndrome-associated peptide, *sSS* Secondary Sjögren’s syndrome

To identify the target antigen of the anti-3S-P antibody in patients with sSS, we prepared an R-anti-3S-P antibody and performed immunohistochemical analysis of synovial and salivary gland tissues from patients with RA-sSS (Additional file 1: Figure S1). We found that the synovial lining layer and ductal cells of the salivary glands from all patients were strongly stained by the R-anti-3S-P antibody (Fig. 2a) and that the target recognized by R-anti-3S-P was present in the cytoplasm of cultured FLSs (Fig. 2b).

### Vimentin is autoantigen in sSS

**Fig. 3:**
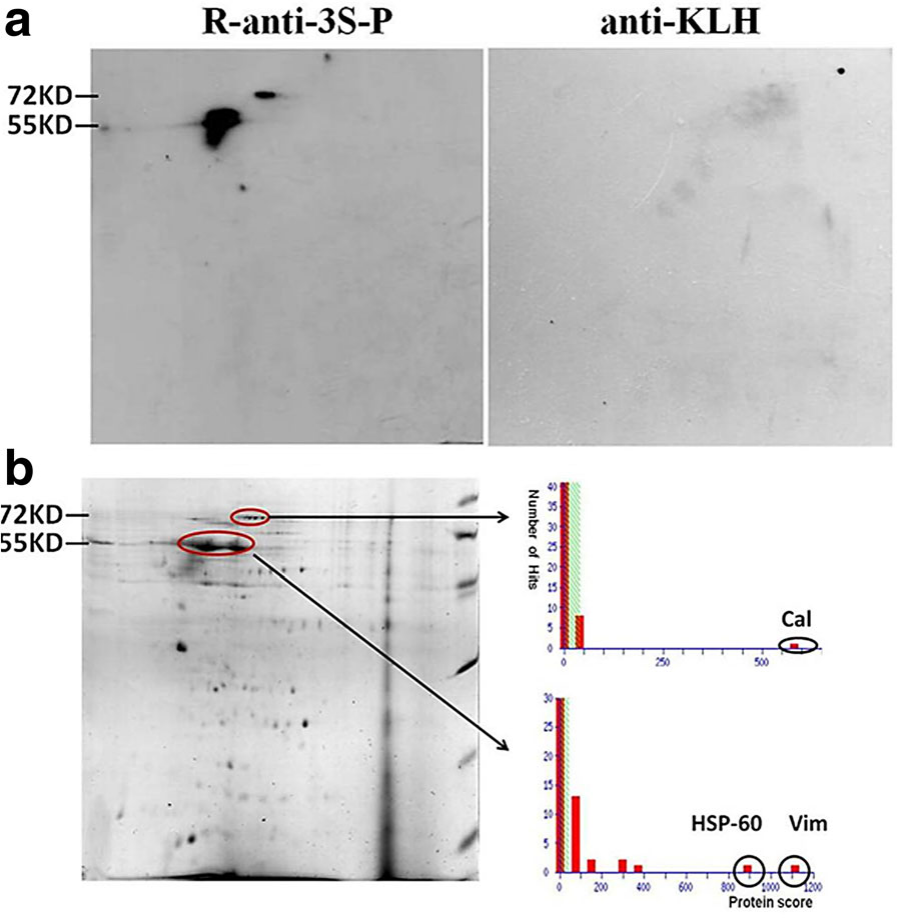
Two-dimensional PAGE of total proteins extracted from FLSs of patients with RA-sSS and Western blot analysis. **a** Western blot showing immunostaining of FLS proteins with R-anti-3S-P and anti-KLH, respectively. **b** Colloidal Coomassie Blue staining of two-dimensional gel of FLS proteins. Protein spots indicated by *circles* were further identified using matrix-assisted laser desorption/ionization time-of-flight MS. Mascot search results showed protein scores of 580, 1113, and 343 for human caldesmon isoform 2 (Cal), vimentin (Vim), and 60 kDa heat shock protein (HSP-60), respectively. *FLS* Fibroblast-like synoviocyte, *KLH* Keyhole limpet hemocyanin, *RA* Rheumatoid arthritis, *R-anti-3S-P* Rabbit-anti-secondary Sjögren’s syndrome-associated peptide, *sSS* Secondary Sjögren’s syndrome

Total proteins extracted from FLSs were analyzed using two-dimensional gel electrophoresis. Compared with control anti-KLH, two spots were recognized by R-anti-3S-P in Western blot analysis (Fig. 3a). The proteins in the two spots were identified as vimentin, heat shock protein 60 (HSP60), and caldesmon isoform 2 using MS analysis. Vimentin and HSP60 were identified from the spots at 55 kDa, and caldesmon isoform 2 was identified from the spot at 72 kDa (Fig. 3b).

**Fig. 4:**
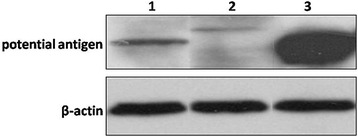
Western blot analysis of proteins extracted from FLSs using antibodies against caldesmon (*lane 1*), HSP60 (*lane 2*), and antivimentin (*lane 3*) as probes. β-Actin was used as an internal control. *FLS* Fibroblast-like synoviocyte, *HSP60* Heat shock protein 60

**Fig. 5:**
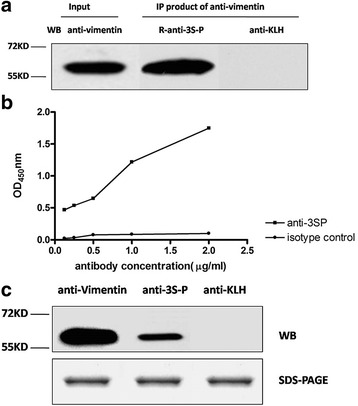
Identification of vimentin as the target autoantigen of anti-3S-P autoantibodies. **a** Autoantigens of anti-3S-P and vimentin are present in immunoprecipitates (IP) obtained by using antivimentin antibody from lysates of FLSs, whereas no band was seen in negative controls. **b** ELISA data for the detection of recombinant human vimentin using the R-anti-3S-P antibody diluted 1:50 to 1:800. The anti-KLH antibody was used as an isotype control and was diluted 1:50 to 1:800. **c** Western blot (WB) of recombinant human vimentin probed with antivimentin, R-anti-3S-P, and anti-KLH antibodies. *ELISA* Enzyme-linked immunosorbent assay, *FLS* Fibroblast-like synoviocyte, *KLH* Keyhole limpet hemocyanin, *R-anti-3S-P* Rabbit-anti-secondary Sjögren’s syndrome-associated peptide, *SDS* Sodium dodecyl sulfate

To further confirm the results derived by MS, we performed immunoprecipitation to pull down target proteins using antibodies against vimentin, HSP60, and caldesmon isoform 2. Western blot analysis revealed that each protein was detected by the cognate respective antibody in extracts of FLSs (Fig. 4). However, R-anti-3S-P recognized the protein precipitated by the antivimentin antibody, not by anti-KLH (Fig. 5a).

**Fig. 6:**
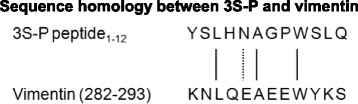
Peptide sequence homologies. Identity is indicated by *solid lines*, and a conservative substitution is indicated by the *dotted line*

Using BLASRP software from the BLAST network service of the NCBI, we found that the sequence of 3S-P is highly similar to that of amino acid residues 282–293 of vimentin (Fig. 6). Moreover, the R-anti-3S-P antibodies detected recombinant human vimentin in ELISA (Fig. 5b) and Western blotting (Fig. 5c) experiments.

## Discussion

In clinical practice, there is no specific serologic marker for sSS, and the immunopathology of this condition is poorly understood. Diagnosis relies on salivary gland biopsy, which is invasive, requires evaluation by an expert histopathologist, and may have adverse sequelae [26]. We report the detection of an antibody against 3S-P in 60 of 88 of patients with sSS in the present study. This assay was highly sensitive and specific for distinguishing sSS from pSS. Furthermore, the profile of reactivity of anti-3S-P differs from those reported for anti-SSA and anti-SSB [14, 15], which suggests that the antigen recognized by anti-3S-P differs from those recognized by anti-SSA and anti-SSB. Taken together, these results indicate that the immunopathogenesis of sSS differs from that of pSS.

In the present study, we determined that vimentin is an autoantigen recognized by anti-3S-P. In humans, vimentin is expressed by a wide variety of mesenchymal cell types, such as fibroblasts and endothelial cells, and is easily detected in the synovium [27, 28]. Antibodies against citrullinated vimentin are prominent in the development of RA [29–32]. Our results show that the antibody present in the majority of patients with sSS tested, which we designated as anti-3S-P, reacted with noncitrullinated recombinant human vimentin. This indicates that the production of anti-3S-P antibodies in patients with sSS may result from epitope spreading, which occurs in several models of autoimmunity [33–36]. There is no evidence, to our knowledge, that antivimentin antibodies are present in patients with SLE. Therefore, the occurrence and clinical correlates of anti-3S-P antibodies in patients with SLE should be investigated further.

## Conclusions

In the present study, we identified an antibody that is highly specific and sensitive for sSS. Further studies are required to determine whether this peptide antibody is useful for clinical diagnosis of sSS and involved in disease pathogenesis. We have demonstrated a novel autoantibody that is highly specific in the diagnosis of sSS and that vimentin is an autoantigen recognized by anti-3S-P. The underlying molecular mechanism of the disease might be epitope spreading involved with vimentin.

## Additional file

Additional file 1: Table S1. Clinical and serological data comparing RA with and without sSS. **Table S2.** Clinical and serological data comparing sSS and pSS. **Table S.** Characteristics of the patients used for library screening. **Figure S1.** Immunohistochemical analysis of tissues and cells using anti-3S-P antibodies. (DOCX 237 kb)

## Abbreviations

3S-P: Secondary Sjögren’s syndrome-associated peptide
AS: Ankylosing spondylitis
BSA: Bovine serum albumin
DAPI: 4′,6-Diamidino-2-phenylindole
ELISA: Enzyme-linked immunosorbent assay
FITC: Fluorescein isothiocyanate
FLS: Fibroblast-like synoviocyte
HC: Healthy control subject
HSP60: Heat shock protein 60
IEF: Isoelectric focusing
Igs: Immunoglobulins
KLH: Keyhole limpet hemocyanin
NCBI: National Center for Biotechnology information
OD: Optical density
PBST: PBS with Tween 20
PEG: Polyethylene glycol
pSS: Primary Sjögren’s syndrome
RA: Rheumatoid arthritis
R-anti-3S-P: Rabbit-anti-secondary Sjögren’s syndrome-associated peptide
SDS: Sodium dodecyl sulfate
SLE: Systemic lupus erythematosus
SS: Sjögren’s syndrome
sSS: Secondary Sjögren’s syndrome
TOF: Time of flight
