# Supplementary material for: Identification of a novel autoantibody against self-vimentin specific in secondary Sjögren’s syndrome
Source: Arthritis Res Ther. 2018 Feb 12;20:30. doi: 10.1186/s13075-017-1508-5 (PMC5810024; doi:10.1186/s13075-017-1508-5)
Supplement: Supplementary file 1 — Clinical and serological data comparing RA with and without sSS. Table S2. Clinical and serological data comparing sSS and pSS. Table S. Characteristics of the patients used for library screening. Figure S1. Immunohistochemical analysis of tissues and cells using anti-3S-P antibodies. (DOCX 237 kb) [file 13075_2017_1508_MOESM1_ESM.docx]

Table S1. Clinical and serological data comparing RA with and without sSS

|  | RA-sSS  n=71(SD) | RA  n=79(SD) | *P* |
| --- | --- | --- | --- |
| Disease duration, yrs | 9.4(6.9) | 9.3(8.7) | 0.98 |
| Number of swollen joints  Per patient | 7.6(8.4) | 5.9(5.5) | 0.45 |
| Number of tender joints  Per patient | 10.3(10.3) | 9.1(10.7) | 0.72 |
| DAS-28 score | 5.4(1.8) | 5.2(1.5) | 0.67 |
| ESR(mm/h) | 62.5(28.8) | 70.9(34.7) | 0.41 |
| CRP(mg/L) | 39.1(37.4) | 58.5(49.9) | 0.17 |
| Anti-CCP(U/ml) | 139.8(80.1) | 160.5(66.3) | 0.40 |
| RF(U/ml) | 390.2(465.9) | 307.6(495.0) | 0.59 |

Table S2. Clinical and serological data comparing sSS and pSS

|  | sSS  n=88 | pSS  n=55 | *P* |
| --- | --- | --- | --- |
| Clinical patterns of SS, n(%) |  |  |  |
| Subjective xerostomia only | 56(47.7) | 37(39.1) | 0.79 |
| Subjective xerophtalmia only | 69(78.4) | 41(74.5) | 0.56 |
| Objective xerostomia^a^ | 35(39.8) | 31(56.4) | 0.07 |
| Objective xerophtalmia^b^ | 61(69.3) | 36(65.5) | 0.77 |
| Serological patterns, n(%) |  |  |  |
| Anti-Ro/SSA antibodies | 41(46.6) | 32(58.2) | 0.23 |
| Anti-La/SSB antibodies | 35(39.8) | 28(51.0) | 0.25 |
| Positive rheumatoid factor | 42(47.7) | 33(60.0) | 0.20 |
| Salivary gland biopsy  % with grade 3 or 4^c^ | 69.3 | 65.5 | 0.76 |

^a^Defined as an unstimulated salivary flow less than 0.1ml/min or an abnormal patotid sialography of an abnormal scintigraphy result of salivary glands with ^99-m^Tc.

^b^Defined as an abnormal Schirmer’s test results (≦5mm in 5min) or Van Bijsterveld score ≥4 after Lissamine green coloration.

^c^According to Chisholm and Mason classification.

Table S3. Characteristics of the patients used for library screening

|  | RA-sSS | RA | HC | P |
| --- | --- | --- | --- | --- |
| Mean age(years) | 52.3 | 49.1 | 50.2 | NS |
| Female/Male | 9/1 | 8/2 | 8/2 | NS |
| Disease duration | 12.3（15.8） | 11.3（8.7） | NA | 0.85 |
| Number of swollen joints  Per patient | 8.2(10.3) | 4.8(7.0) | NA | 0.40 |
| Number of tender joints  Per patient | 9.1(12.1) | 9.0(9.8) | NA | 0.98 |
| DAS-28 score | 5.3(1.6) | 5.1(1.5) | NA | 0.72 |
| ESR(mm/h) | 56.1(34.5) | 85.7(29.4) | NA | 0.05 |
| CRP(mg/L) | 46.8(43.3) | 70.3(55.4) | NA | 0.31 |
| Anti-CCP(U/ml) | 137.3(75.0) | 186.7(46.1) | NA | 0.13 |
| RF(U/ml) | 121.0(113.8) | 119.4(104.5) | NA | 0.97 |

NA: not applicable

*
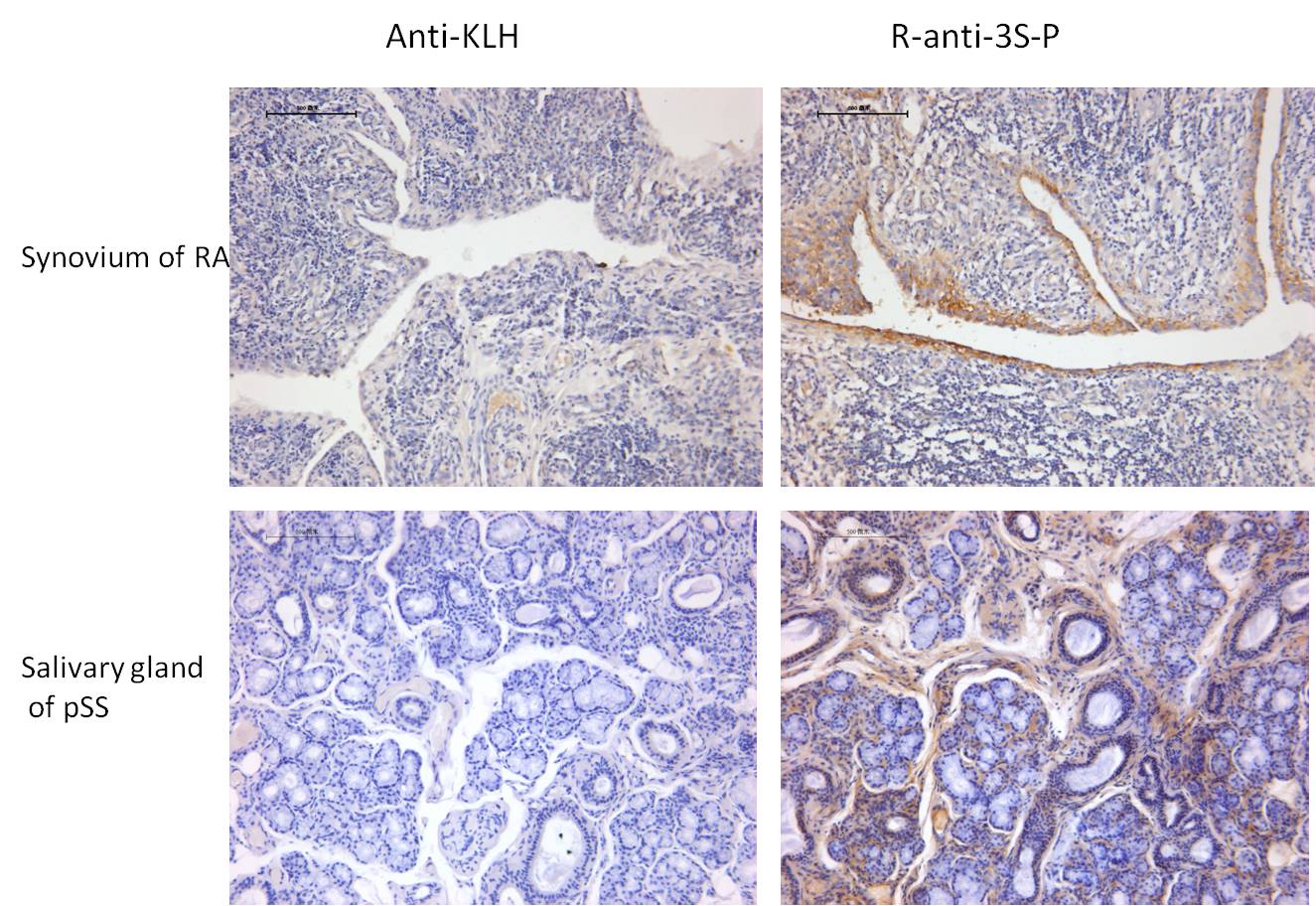
*

Fig S1. Immunohistochemical analysis of tissues and cells using anti-3S-P antibodies. Figure shows the expression of anti-3S-P target antigens in RA synovial tissue and pSS salivary tissue. RA synovium was stained using antibodies against KLH and R-anti-3S-P. pSS salivary gland tissue was stained using R-anti-3S-P and anti-KLH as control (original magnification × 400).
